# Improved clinical outcomes of preimplantation genetic testing for aneuploidy using MALBAC-NGS compared with MDA-SNP array

**DOI:** 10.1186/s12884-020-03082-9

**Published:** 2020-07-03

**Authors:** Wenbin Niu, Linlin Wang, Jiawei Xu, Ying Li, Hao Shi, Gang Li, Haixia Jin, Wenyan Song, Fang Wang, Yingpu Sun

**Affiliations:** 1 Center for Reproductive Medicine, The First Affiliated Hospital of Zhengzhou University, Eastern Jianshe Road, Erqi District, Zhengzhou City, Henan Province People’s Republic of China; 2 Henan Key Laboratory of Reproduction and Genetics, The First Affiliated Hospital of Zhengzhou University, Eastern Jianshe Road, Erqi District, Zhengzhou City, Henan Province People’s Republic of China

**Keywords:** Preimplantation genetic testing for aneuploidy, Single nucleotide polymorphism, Next generation sequencing, Pregnancy outcome

## Abstract

**Background:**

To assess whether preimplantation genetic testing for aneuploidy with next generation sequencing (NGS) outweighs single nucleotide polymorphism (SNP) array in improving clinical outcomes.

**Methods:**

A retrospective analysis of the clinical outcomes of patients who underwent PGT-A treatment in a single center from January 2013 to December 2017.A total of 1418 couples who underwent PGT-A treatment were enrolled, of which 805 couples used NGS for PGT-A, while the remaining 613 couples used SNP array for PGT-A. Clinical pregnancy rate, miscarriage rate and healthy baby rate were compared between the MALBAC-NGS-PGT-A and MDA-SNP-PGT-A groups.

**Results:**

After testing karyotypes of 5771 biopsied blastocysts, 32.2% (1861/5771) were identified as chromosomally normal, while 67.8% were chromosomally abnormal. In terms of clinical outcomes, women in the MALBAC-NGS-PGT-A group had a significantly higher clinical pregnancy rate (50.5% vs 41.7%, *p* = 0.002) and healthy baby rate (39.6% vs 31.4%, *p* = 0.003), and a lower miscarriage rate (15.5% vs 22.8%, *p* = 0.036).

**Conclusion:**

This is the largest study reporting the extensive application of NGS-based PGT-A, whilst comparing the clinical outcomes of MALBAC-NGS-PGT-A and MDA-SNP-PGT-A. The results provide greater evidence supporting the wider use of NGS in PGT-A, not only for its lower cost but also for its improved clinical outcomes compared to SNP-based PGT-A.

## Background

Estimates have indicated that only one-third of natural conceptions progress to a healthy live birth [1, 2]. Even after far advanced assisted reproductive technology (ART) was introduced for the management of many kinds of subfertility, the healthy baby rate for embryo transfer cycles was still reported to be less than 30% [3]. Fetal chromosomal aberrations have been recognized as the leading cause of the poor healthy live birth rate, and account for up to 70% of miscarriages [4]. Additionally, various chromosomal abnormalities can be detected in both younger and older women [4]. A study even found that more than 90% of blastomeres in human preimplantation embryos had at least one chromosomal abnormality in one or more cells [5]. Therefore, in theory, transferring chromosomally normal embryos should be an effective way to reduce the miscarriage rate as well as to increase the pregnancy rate.

Preimplantation genetic testing for aneuploidy (PGT-A) has been used for decades to minimize chromosomally abnormal pregnancies and to select chromosomally normal embryos prior to transfer, which is reported to have remarkably reduced the risk of an affected pregnancy and miscarriage [3, 6, 7]. This treatment benefits couples with chromosome abnormalities, repeated IVF failures (occurring more than three times) and those with advanced maternal age (over 35 years) [8].

Fluorescence in situ hybridization (FISH) was the first molecular cytogenetic technique widely used in PGT-A treatment but the limited number of available fluorescent probes targeted at whole chromosomes, and complex sample preparation procedures, restricted its application [9]. In contrast, array-based genome-wide techniques such as single nucleotide polymorphism (SNP) microarray allow every chromosome to be evaluated simultaneously [10]. Studies have shown that array-based PGT-A treatment may increase the pregnancy rate to 69.4% compared to FISH which has a pregnancy rate of 38.4% [11]. However, its relatively high cost may restrict its usage in clinical practice.

Recent advances in NGS are increasing its range of application in PGT-A clinical practice. The accuracy of NGS in detecting chromosomally abnormal embryos has been extensively reported [12–14]. Both NGS and SNP array rely on whole genome amplification (WGA) for a single-cell biopsy to generate enough DNA. The most common WGA methods are multiple displacement amplification (MDA) for SNP array and multiple annealing and looping-based amplification cycles (MALBAC) for NGS. Studies have suggested that MDA and MALBAC possess similar single-nucleotide variant detection, false-positive rates and allelic dropout rates [15].

However, very few studies have reported the clinical outcomes of NGS-based PGT-A treatment, therefore this study compares clinical outcomes between NGS-based and SNP array-based PGT-A, including the clinical pregnancy rate, miscarriage rate and healthy baby rate, to contribute to this field of research.

## Methods

### Patients

This study was approved by the Ethics Committee of The First Affiliated Hospital of Zhengzhou University. Between January 2013 and December 2017, a total of 1418 couples received PGT-A treatment after being evaluated by both a geneticist and an infertility specialist in the reproductive center of The First Affiliated Hospital of Zhengzhou University. Factors leading to PGT-A treatment included abnormal karyotypes, advanced maternal age (AMA≥35 years), repetitive implantation failures (RIF ≥ 3times) and/or recurrent miscarriage (RM ≥ 2times). To avoid DNA contamination from the paternal genome, all PGT-A patients underwent intracytoplasmic sperm injection (ICSI) treatment irrespective of their sperm quality.

The patients were divided into two main groups: MALBAC-NGS-PGT-A group and MDA-SNP-PGT-A group. Each group was subsequently classified into five subgroups according to the treatment indications of couples: Robertsonian translocation, reciprocal translocation, inversion, sex chromosome abnormality karyotype and undergoing PGS (patients with AMA or RIF or RM) treatment.

In the MALBAC-NGS-PGT-A group, a total of 805 couples were treated between January 2016 and December 2017: 157 with a Robertsonian translocation, 405 with a reciprocal translocation, 40 with an inversion, 92 with a sex chromosome abnormality karyotype and 111 with PGS treatment indications. Among the couples undergoing PGS treatment,54 were of advanced maternal age, 47 had experienced recurrent miscarriages and 10 were patients with repeated implantation failures.

In the MDA-SNP-PGT-A group, 613 couples were treated from January 2013 to December 2015. Five hundred nineteen couples were found to be chromosomally abnormal. Among them, the number of couples with a Robertsonian translocation, reciprocal translocation, inversion and sex chromosome abnormality karyotype were 105, 342, 14 and 58 respectively. The remaining 94 couples underwent PGS treatment due to advanced maternal age (39 couples), recurrent miscarriages (46 couples) and repeated implantation failures (9 couples).

### Controlled ovarian stimulation, embryo biopsy and transfer

Controlled ovarian stimulation (COS) was performed using the standard long protocol. Gonadotropin-releasing hormone agonist (GnRH-a), Decapeptyl (Germany) or Diphereline (France) were used to induce complete down-regulation (follicle stimulating hormone FSH ≤ 5 IU/L, luteinizing hormone LH ≤ 5 IU/L, estrogen E2 ≤ 50 pg/ml, diameter of the largest follicle≤10 mm and endometrial thickness ≤ 5 mm). Gonal-F (Gn, Switzerland) was subsequently administered. The starting dose of Gn was determined based on body mass index, age, basal FSH level and antral follicle count. Where the diameter of the dominant follicle was more than 14 mm, fasting blood was collected every day to determine the levels of LH, E2 and P present. The regimen of Gn was adjusted based on the hormone levels and follicle size. When the amount of follicles with a diameter ≥ 16 mm was more than two-thirds, and the diameter of the dominant follicle was greater than 20 mm, human chorionic gonadotropin was intramuscularly injected. Thirty-seven hours later, oocyte retrieval was performed using ultrasonic guidance. Fertilization took place 3–4 h after oocyte retrieval. Embryo cleavage was recorded every 24 h. Blastocysts were graded on the fifth or sixth day after oocyte retrieval using the Gardner criteria. Trophectoderm (TE) biopsy was performed on selected blastocysts higher than 3BB, and 5 to 10 TE cells were isolated and cut using a laser. The biopsied cells were then immediately placed in the RNAse and DNAse-free polymerase chain reaction tube and transported to our PGT-A center. After testing karyotypes of the biopsied blastocysts, a single thawed euploid embryo was transferred for each transfer cycle.

### NGS testing and data analysis

MALBAC was used for WGA in NGS testing, following the manufacturer’s protocol (XK028,Yikon Genomics, China). Amplified DNA was then diluted, fragmented, adaptor ligated, subjected to PCR reaction and purified. After measuring the concentration, libraries were processed using rapid single-end 50 cycle mode sequencing using a Hiseq 2500 sequencer (Illumina, USA) in our center.

The sequenced raw data were demultiplexed and converted to the FASTQ format using CASAVA 1.8.4(Illumina, USA).MALBAC primers, adaptors and low-quality bases were removed from the FASTQ data using Trimmomatic [16],generating an average of ~ 2 million filtered reads per sample, with an average ~ 0.03 × sequencing depth.

High-quality reads were mapped to a hg19 reference genome using the Burrows-Wheeler Aligner software package [17]. Unique mapped reads were extracted from the alignment reads (bam file). The reference genome was then divided into non-overlapping observation windows (bins) with a size of 1000Kb. The number of reads and GC-content were calculated in each bin and GC bias correction was performed for every 1% GC-content [18]. The R programming language was then used to graph the final relative number of reads for each bin to visualize copy number variations.

### SNP array and data analysis

MDA was performed using REPLI-g Single Cell Kit (150,345, QIAGEN, Germany) in SNP array according to the authors’ previous report [19]. Briefly, biopsied TE cells (5 to 10 cells) were lysed in DTT and DLB buffer. Whole genome DNA was amplified in an amplification buffer created by mixing REPLI-g single-cell DNA polymerase and the reaction buffer. The MDA product was then hybridized on the Illumina HumanCyto12 microarray by following the user manual. Data were analyzed using GenomeStudio Software (2011, Illumina, USA). The genotype was evaluated by using the B allele frequency and log_2_R ratio (log_2_R = Log_2_ normalized *R* value/expected normalized *R* value).

### Clinical outcomes and statistical analysis

Chromosomally normal/balanced blastocysts were transferred. 35 to 45 days later, clinical pregnancy was confirmed by the results of an ultrasound examination. Clinical pregnancy rate (clinical pregnancy cycles/embryo transferred cycles), miscarriage rate (embryo lost cycles/clinical pregnancy cycles) and healthy baby rate (healthy babies/ number of transferred embryos) were recorded as the main outcomes. SPSS 21.0 (IBM, USA) was used for statistical analysis. Analysis of the maternal age, BMI, basal FSH level and endometrial thickness was made using Student’s t-test. Pearson’s chi-squared test was also used to analyze categorical data. A *P*-value≤0.05 was considered significant.

## Results

### Results of NGS tested blastocysts

**Fig. 1:**
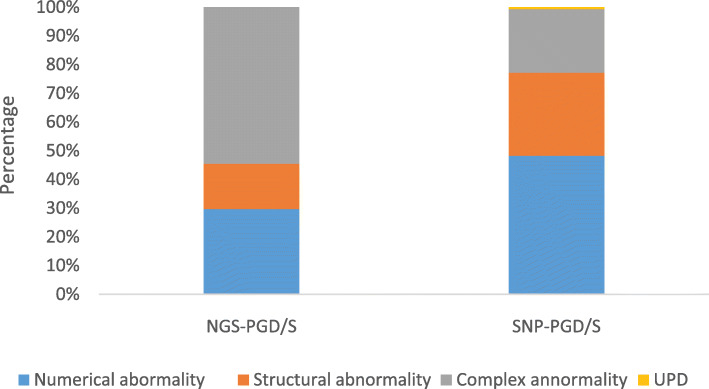
The distribution of chromosomal abnormalities in NGS-PGD/S and SNP-PGD/S groups

NGS testing was successfully performed on 805 couples and 3321 biopsied blastocysts. As is shown in Tables 1, 766 (23.1%) euploid/normal and 2555(76.9%)chromosomally abnormal samples were identified. Of these abnormalities, 760(22.9%) had numerical abnormalities, 404(12.2%)contained structural abnormalities (deletions and duplications), while 1391(41.8%) had complex abnormality (defined as two or more duplications/deletions involving the same, or two or more chromosomes) (Fig. 1).

**Table 1:** NGS testing for blastocysts

Clinical data	Couples					Total
	Robertsonian translocation	Reciprocal translocation	Inversion	PGS	Sex chromosome abnormality	
No. of cases	157	405	40	111	92	805
Maternal age (mean ± SD)	30.2 ± 4.9	29.4 ± 4.09	30.0 ± 4.03	35.4 ± 5.35	28.7 ± 4.06	30.3 ± 4.76
No. of blastocysts biopsied	603	1825	153	395	345	3321
Euploidy	164 (27.2%)	313 (17.2%)	54 (35.3%)	130 (32.9%)	105 (30.4%)	766 (23.1%)
Numerical abnormality	264 (43.8%)	241 (13.2%)	29 (18.9%)	131 (33.2%)	95 (27.5%)	760 (22.9%)
Structural abnormality	41 (6.8%)	237 (13.0%)	22 (14.4%)	42 (10.6%)	62 (18.0%)	404 (12.2%)
Complex abnormality	134 (22.2%)	1034 (56.6%)	48 (31.4%)	92 (23.3%)	83 (24.1%)	1391 (41.8%)

**Fig. 2:**
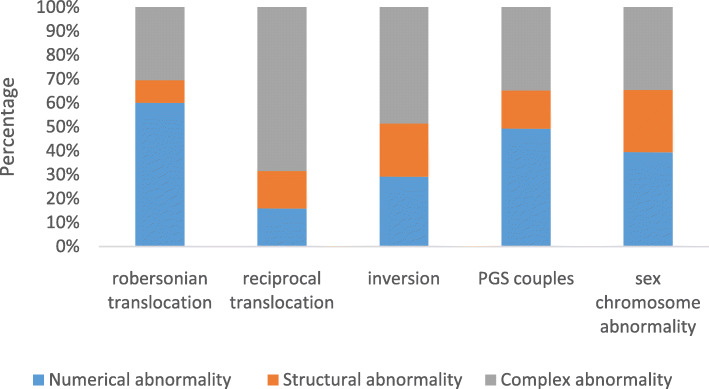
The distribution of chromosomal abnormalities in NGS-PGD/S subgroups

The most common chromosome abnormality type in each subgroup was also determined, as shown in Fig. 2. Numerical abnormality was most frequently found in blastocysts from couples with Robertsonian translocation (43.8%, 264/603), sex chromosome abnormality (27.5%, 95/345) and PGS couples (33.2%, 131/395), while structural abnormality showed a relatively balanced distribution among the five subgroups. Meanwhile, complex abnormality was more common than the other kinds of abnormalities for couples with reciprocal translocation (56.6%, 1034/1825) and inversion (31.4%, 48/153) (Table 1).

### Results of SNP array tested blastocysts

Among the 2450 blastocysts tested by SNP array from 613 couples, 1095 (44.7%) were euploid/normal and 1355(55.3%) were abnormal. Among all of the detected abnormalities, 654 (26.7%) had a numerical abnormality, 393 (16%) had a structural abnormality (duplication or deletion), 299 (12.2%) contained complex abnormalities, and the remaining 9 (0.4%) showed uniparental disomy (UPD) (Fig. 1).

**Fig. 3:**
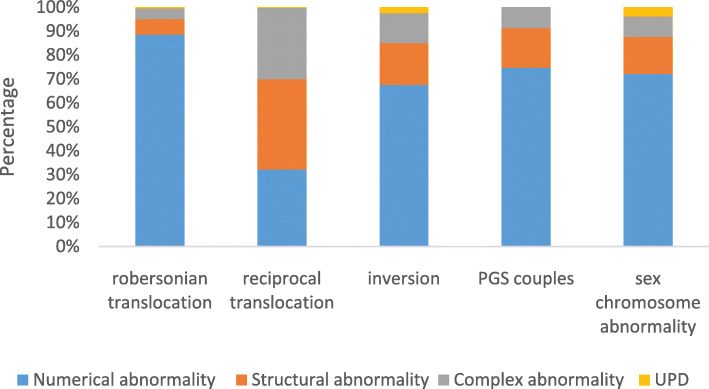
The distribution of chromosomal abnormalities in SNP-PGD/S subgroups

With regard to the distribution of chromosomal aberration types among each subgroup, numerical abnormality was most commonly found in samples from couples with Robertsonian translocation, inversion, PGS and sex chromosome abnormality, accounting for 38.7% (177/457), 32.9% (27/82), 28.3% (86/303) and 34.9%(75/215) for each of the above, respectively. Among couples with reciprocal translocation, structural abnormality (24.3%, 338/1393) was identified to be the most frequent (Table 2 and Fig. 3).

**Table 2:** SNP array testing for blastocysts

Clinical data	Couples					Total
	Robertsonian translocation	Reciprocal translocation	Inversion	PGS	Sex chromosome abnormality	
No. of cases	105	342	14	94	58	613
Maternal age (mean ± SD)	29.7 ± 4.18	29.1 ± 4.24	30.9 ± 4.08	33.9 ± 4.97	29.7 ± 4.29	29.9 ± 4.68
No.of blastocysts biopsied	457	1393	82	303	215	2450
Euploidy(%)	257 (56.2%)	497 (35.7%)	42 (51.2%)	188 (62.0%)	111 (51.6%)	1095 (44.7%)
Numerical abnormality (%)	177 (38.7%)	289 (20.7%)	27 (32.9%)	86 (28.3%)	75 (34.9%)	654 (26.7%)
Structural abnormality (%)	13 (2.8%)	338 (24.3%)	7 (8.5%)	19 (6.3%)	16 (7.4%)	393 (16%)
Complex abnormalities(%)	9 (2.0%)	266 (19.1%)	5 (6.0%)	10 (3.3%)	9 (4.2%)	299 (12.2%)
UPD (%)	1 (0.2%)	3 (0.2%)	1 (1.2%)	0	4 (1.9%)	9 (0.4%)

### Clinical outcomes

Among the 805 couples in the MALBAC-NGS-PGT-A group, 270 had no euploid/normal embryos to transfer, 522 underwent embryo transfer, and 13 are still waiting for transfer. Of the 522 couples with embryo transfer, 675 euploid/normal embryos were transferred in 675 cycles. Clinical pregnancy was identified in 341 (50.5%) of the transferred cycles. Healthy baby rate was 39.6% (267/675,including two onset of twins). Of the clinical pregnancy cycles, 15.5%(54/341) of the cycles ended with miscarriage. In the SNP array-PGT-A group, 145 couples were found with no euploid embryos to transfer, while 468 couples had euploid/normal embryos. To date, 452 couples have undergone embryo transfer while 12 couples were excluded in this study because they transfered two embryos in once transfer cycle, and 16 couples are still waiting for transfer. Of the 440 couples with embryo transfer, 547 euploid/normal embryos were transferred in 547 cycles. Of the 547 transferred cycles, 41.7% (228/547) of the cycles resulted in clinical pregnancy, healthy baby rate was 31.4% (172/547,including two onset of twins). Of the clinical pregnancy cycles, 22.8% (52/228) of these miscarried.

There were no significant differences in terms of maternal age, BMI, basal FSH level and endometrial thickness on the day of embryo transfer between the MALBAC-NGS-PGT-A and MDA-SNP-PGT-A groups (*P* > 0.05). However, a significantly higher clinical pregnancy rate (50.5% vs41.7%, *P* = 0.002), healthy baby rate (39.6% vs31.4%, *P* = 0.003) and lower miscarriage rate (15.5% vs22.8%, *P* = 0.036) were observed in the MALBAC-NGS-PGT-A group when compared with the MDA-SNP-PGT-A group (Table 3).

**Table 3:** Clinical outcomes in NGS-PGT-A group and SNP-PGT-A group

Clinical measures	NGS based PGT-A	SNP array based PGT-A	*P*-value
No. of couples participating	805	613	
No. of couples with transfer	522	440	
No. of couples without available embryos	270	145	
No. of embryos transferred	675	547	
Transferred cycles	675	547	
Maternal age (mean ± SD) of couples with transfer	29.97 ± 4.26	29.78 ± 4.42	0.489
PGT-A indications			
Robertsonian translocation	21.3%(111/522)	19.5%(86/440)	0.510
Reciprocal translocation	44.6%(233/522)	53.4%(235/440)	0.007*
Inversion	6.1%(32/522)	2.5%(11/440)	0.007*
Sex chromosome abnormality	13.8%(72/522)	8.6%(38/440)	0.012*
AMA	5.0%(26/522)	4.5%(20/440)	0.753
RM	7.5%(39/522)	9.5%(42/440)	0.248
RIF	1.7%(9/522)	1.8%(8/440)	0.912
Maternal BMI (mean ± SD) of couples with transfer	22.76 ± 3.00	22.70 ± 2.96	0.799
Basal FSH level (mean + SD) of women with transfer	6.51 ± 1.88	6.71 ± 1.80	0.113
Endometrial thickness (mean + SD) ^a^	9.8 ± 1.91	9.7 ± 1.94	0.544
Clinical pregnancy rate^b^	50.5%(341/675)	41.7%(228/547)	0.002*
Miscarriage rate ^c^	15.5%(54/341)	22.8%(52/228)	0.036*
Healthy baby rate^d^	39.6%(267/675)	31.4%(172/547)	0.003*

^a^Endometrial thickness measured at embryos transferred day

^b^Clinical pregnancy rate = clinical pregnancy cycles /embryo transferred cycles

^c^Miscarriage rate = embryo lost cycles /clinical pregnancy cycles

^d^Healthy baby rate = healthy babies/ number of transferred embryos

*p < 0.05, statistical significance

## Discussion

The study retrospectively analyzed the biopsy results of 5771 blastocysts to evaluate the clinical outcomes of NGS-based PGT-A treatment. A comparison between the clinical outcomes of women in the MALBAC-NGS-PGT-A and MDA-SNP-PGT-A groups showed that the MALBAC-NGS-PGT-A group had a significantly higher clinical pregnancy rate, healthy baby rate and lower miscarriage rate, supporting the application of NGS-based PGT-A treatment to in patients with chromosomal abnormalities, advanced maternal age, repeated IVF failures and/or recurrent miscarriage.

After testing the karyotypes of a total of 5771 biopsied blastocysts, only 32.2% (1861/5771) of embryos were identified as chromosomally normal, and more than half of the embryos were found with various types of chromosomal abnormalities present, indicating the necessity of normal/euploid embryo selection, especially for couples with abnormal karyotypes. Since the first successful birth using NGS-based PGD/PGS in 2013 in Philadelphia [12], several studies have demonstrated the availability and efficiency of using NGS in PGT-A therapy. Fiorentino et al. performed a large preclinical and blind study to validate the accuracy of NGS-based whole chromosome aneuploidy screening [13]. By comparing the embryos obtained with previously established arrayCGH methodology, they confirmed that NGS has a 100% aneuploidy diagnosis consistency with arrayCGH. In addition to aneuploidy screening, Sachdeva et al. reported the ability of NGS to detect segmental changes (as small as 14 Mb in size) [20], suggesting it could also be applied in the diagnosis of partial aneuploidy. Furthermore, by comparing NGS results with two other independent methodologies, namely qPCR-based comprehensive chromosome screening and Taqman allelic discrimination assays, Treff et al. validated the use of NGS in PGD treatment for patients with monogenic disease [21].

The current study has not only validated NGS as a reliable technique in selecting chromosomally normal/euploid embryos for transfer, but has also provided evidence of better clinical outcomes for women using NGS-based PGT-A than for those using SNP-based PGT-A. This could be partially explained by the difference in accuracy in detecting chromosomal abnormalities between these two techniques. Tan et al. compared the accuracy by testing 150 blastocysts using both NGS and SNP array [9]. Their results showed that all the tests of normal/balanced blastocysts in the NGS group produced results consistent with those using SNP array, but when it came to chromosomally abnormal blastocysts, seven cases were found to be inconsistent. Further validation was sought by testing the blastocysts using qPCR. This produced results consistent with the NGS tests, indicating that NGS has a higher resolution for chromosomal abnormality detection.

Furthermore, Yin et al. examined 38 blastocysts using NGS and compared the results with those results obtained using SNP array [22]. They demonstrated that both NGS and SNP array could detect embryo aneuploidy with 100% consistency, but NGS provided higher accuracy in some areas for embryos with unbalanced chromosomal rearrangement [22], likely due to its ability to correct the WGA bias during data analysis. Therefore, the lower accuracy and resolution of SNP array in detecting chromosomally abnormal embryos may result in incorrectly categorizing chromosomally abnormal embryos as being chromosomally normal, leading to their transfer with chromosomal abnormal embryos, and resulting in a miscarriage.

In addition, recent studies have shown that the use of NGS to detect mosaicism can be carried out to a much greater sensitivity than SNP array [23, 24]. By using SNP array, if ideal results are obtained, mosaicism associated with proportions of aneuploid cells ranging from 40 to 60% could be detected with a high degree of confidence [23]. Proportions of abnormal cells outside this range are indistinguishable between either normality or non-mosaic aneuploidy. In contrast, using NGS, mosaicism could be detected in proportions of abnormal cells ranging from 20 to 80% [25]. Meanwhile, mosaic embryo transfer results in a low implantation rate, pregnancy rate and a higher miscarriage rate compared with chromosomal normal embryo transferring [26]. Thus, the transfer of undetectable mosaic embryos which were falsely identified as normal embryos by SNP array may partly explain the worse clinical outcomes observed in MDA-SNP-PGT-A.

Different WGA methods used in the current study can also be an potential reasons for the different clinical outcomes (MDA for SNP-PGT-A and MALBAC for NGS-PGT-A). MALBAC was found to have a higher genomic coverage, level of specificity, uniformity and reproducibility than MDA in single cell sequencing [27]. However, low-coverage sequencing data reads, as few as 0.1 million, were shown to be able to identify all types of aneuploidy accurately [28]. In addition, recent study found that MALBAC had a higher success rates in detecting all the copy number variants compared with MDA at the single-cell level. While, when five or more cells were used as template, these two methods did not differ significantly [29]. Considering five to ten biopsy cells were used in this study, we hypothesis the effects of different WGA methods on clinical outcomes may not be the main cause of different clinical outcomes observed between MDA-SNP-PGT-A and MALBAC-NGS-PGT-A groups. However, one major limitation of the current study might be that our study cohort was retrospectively established, leaving potential confounding factors un-estimated. A prospective, multi-center randomly controlled study is in need to increase the solidity of our conclusions. Additionally, there are 29 couples still waiting for embryo transfer and 15 couples with ongoing pregnancy, we are unable to obtain the complete pregnancy outcomes and healthy baby rate. This represents a minor limitation of this study, but the relatively large study cohort might alleviate the effects of this limitation exerted on our research conclusions.

Based on the better clinical outcomes indicated in this study, and the continuously decreasing cost of NGS, it is anticipated that NGS will play an important role in PGT-A therapy. The current study is the largest one to date in evaluating MALBAC-NGS-PGT-A clinical outcomes. Our subsequent efforts will be directed at testing the difference in diagnostic accuracy between NGS and SNP array in detecting chromosomal abnormalities in biopsied embryos.

## Conclusion

This is the largest study reporting the extensive application of NGS-based PGT-A and comparing the clinical outcomes of MALBAC-NGS-PGT-A and MDA-SNP-PGT-A. The results provide further evidence supporting the wider use of NGS in PGT-A, not only for its lower cost but also for its better clinical outcomes.

## Data Availability

The data that support the study are available upon reasonable request to the corresponding author.

## Abbreviations

ART: Assisted reproductive technology
PGT-A: Preimplantation genetic testing for aneuploidy
FISH: Fluorescence in situ hybridization
SNP: Single nucleotide polymorphism
NGS: Next generation sequencing
WGA: Whole genome amplification
MDA: Multiple displacement amplification
MALBAC: Multiple annealing and looping-based amplification cycles
COS: Controlled ovarian stimulation
GnRH-a: Gonadotropin releasing hormone agonist
FSH: Follicle stimulating hormone
BMI: Body mass index
UPD: Uniparental disomy
